# High Glucose Represses the Anti-Proliferative and Pro-Apoptotic Effect of Metformin in Triple Negative Breast Cancer Cells

**DOI:** 10.3390/biom9010016

**Published:** 2019-01-08

**Authors:** Sharon Varghese, Samson Mathews Samuel, Elizabeth Varghese, Peter Kubatka, Dietrich Büsselberg

**Affiliations:** 1 Department of Physiology and Biophysics, Weill Cornell Medicine-Qatar, Education City, Qatar Foundation, Doha 24144, Qatar; scv2002@qatar-med.cornell.edu (S.V.); sms2016@qatar-med.cornell.edu (S.M.S.); elv2007@qatar-med.cornell.edu (E.V.); 2 Department of Medical Biology, Jessenius Faculty of Medicine, Comenius University in Bratislava, 03601 Martin, Bratislava, Slovakia; kubatka@jfmed.uniba.sk

**Keywords:** anti-cancer therapy, breast cancer, hyperglycemia/diabetes, metformin, triple negative breast cancers

## Abstract

Metformin, the most widely prescribed anti-diabetic drug, is shown to possess anti-cancer potential in treatment of cancers, including breast cancer; decreases breast cancer risk; and improves overall survival. However, reports suggest that higher glucose concentrations may negatively impact the anti-cancer efficacy of metformin. Therefore, we examined the anti-cancer potential of metformin in triple-negative breast cancer cells (TNBCs) exposed to different glucose (25 mM, 5.5 mM and zero glucose/glucose-starved) conditions. Our data indicates that a high glucose (25 mM) concentration (mimicking diabetes) significantly abrogated the effect of metformin on cell proliferation, cell death and cell cycle arrest in addition to loss of efficacy in inhibition of the mTOR pathway, a key metabolic pathway in TNBC cells. The mTOR pathway is activated in TNBCs compared to other subtypes of breast cancer, regulates the synthesis of proteins that are critical for the growth and survival of cancer cells and its activation is correlated to poor outcomes among TNBC patients, while also contributing to metastatic progression and development of resistance to chemotherapy/radiotherapy. Our studies were performed in two different types of TNBCs, MDA-MB-231 cells (mesenchymal stem cell-like (MSL)) and MDA-MB-468 (basal like-1 (BL-1)). Interestingly, lower concentrations of metformin (50, 100, 250, and 500 μM) significantly increased cell proliferation in 25 mM glucose exposed MDA-MB-231 cells, an effect which was not observed in MDA-MB-468 cells, indicating that the effective concentration of metformin when used as anti-cancer drug in TNBCs may have to be determined based on cell type and blood glucose concentration. Our data indicates that metformin treatment was most effective under zero glucose/glucose-starved conditions in MDA-MB-468 with a significant increase in the apoptotic population (62.3 ± 1.5%; *p*-value < 0.01). Under 5.5 mM glucose conditions in both MDA-MB-231 and MDA-MB-468 cells our data showed reduced viability of 73.56 ± 2.53%; *p*-value < 0.05 and 70.49 ± 1.68%; *p*-value < 0.001, respectively, along with a significant increase in apoptotic populations of both cell types. Furthermore, metformin (2 mM) inhibited the mTOR pathway and its downstream components under zero glucose/glucose-starved conditions indicating that using metformin in combination with agents that inhibit the glycolytic pathway should be more beneficial for the treatment of triple-negative breast cancers in diabetic individuals.

## 1. Introduction

De-regulation of cell energetics and re-programming of energy metabolism is a hallmark of cancer. Cancer cells upregulate glycolysis even in the presence of sufficient oxygen (the Warburg effect), increasing the need for glucose as the key nutrient to maintain key cell processes [1,2,3]. Recent findings indicate that impaired glucose metabolism is an independent risk factor in the development of multiple cancers such as that of the liver, pancreas, kidney and breast in diabetic patients [4]. Therefore, research targeting glucose metabolism as a cancer therapy has gained momentum [1]. Enzymes responsible for key stages of the glycolytic pathway (hexokinase and pyruvate kinase) have emerged as potential targets for chemotherapy [4,5,6]. Thus, chemotherapeutic inhibition of aberrant glycolysis in cancers is an active area of research.

Type 2 diabetes involve multiple metabolic imbalances characterized by dyslipidemia (primarily in the form of increase in non-esterified fatty acids), insulin resistance, hyperinsulinemia, and hyperglycemia amounting to accelerated weight gain, cardiovascular diseases, peripheral vascular diseases [7], and other diseases [4,8]. Retrospective studies showed an association between diabetes and increased incidence risk of colorectal, liver, kidney, and pancreatic cancers [4,9,10] and reinforcing the long-standing link between diabetes and the incidence and progression of breast cancer [4,9,11,12,13].

Metformin is the most widely prescribed oral anti-diabetic drug owing to minimal short and long-term side effects as well as minimal risk of hypoglycemia or weight gain [14,15]. Metformin is also used in the treatment of various other diseases such as obesity [16], infertility, polycystic ovary syndrome (PCOS), and cancers [15]. While insulin and its analogues cause an increased risk in cancers of the pancreas, colo-rectal cancer as well as the breast cancers, metformin has been widely studied for its potential anti-neoplastic efficacy and were linked to decreasing the risk of breast cancer, improving overall survival and sensitizing cancers to conventional chemo- and radiotherapy [4]. Metformin has shown chemotherapeutic effects against breast cancer [11,13,17] and inhibits pro-tumorigenic effects [18]. Treatment with metformin can improve survival and reduce risk of breast cancer [8]. In breast cancers, metformin induced apoptosis through the activation of 5′ adenosine monophosphate-activated protein kinase (AMPK) signaling pathway [19] but other reports have challenged this mechanism [20]. Metformin also reportedly mimics caloric restrictions [21] and also inhibits the growth of endothelial cells, thereby inhibiting tumor angiogenesis [19].

Triple negative breast cancer (TNBC) is aggressive and does not express the estrogen receptor (ER), the progesterone receptor (PR) or the human epidermal growth factor receptor-2 (HER2) and hence cannot be treated using therapeutic approaches that target the ER, PR, or HER2 [22,23]. Although TNBCs are susceptible to chemotherapeutic agents, they develop resistance to chemo- and radiotherapy approaches, metastasize and tend to relapse and is often associated with poor prognosis among affected patients [23,24]. Since TNBCs account for 10–24% of all breast cancers [23] research leading to a successful form of treatment against TNBC is crucial [23]. Deregulation of the mTOR (mammalian target of rapamycin) pathway has been widely reported in TNBCs when compared to ER^+^/PR^+^/HER2 overexpressing breast cancers, and is correlated to the poor outcome among TNBC patients [23]. Increase in phosphorylation and thus activation of the phosphatidylinositol-3-kinase/Akt/mTOR signaling pathways have been reported in TNBC cells [25,26,27,28]. The mTOR pathway is essential for the regulation of synthesis of proteins that are critical for the growth and survival of cancer cells and therefore supports rapid progression of the cell-cycle in TNBCs [28,29]. Furthermore, activation of the mTOR pathway supports metastatic progression and development of resistance to chemotherapy/radiotherapy [28,30]. Targeted inhibition of the mTOR pathway thus can support the treatment of TNBCS, overcome resistance to chemotherapy/radiation therapy and block aggressive phenotypes in these cancers [31]. The anti-neoplastic activity of metformin through AMPK activation via mTORC1 inhibition lead to inhibition of protein synthesis and cell proliferation [32]. However, metformin mediated, AMPK independent inhibition of mTOR has also been reported through REDD1, a hypoxia inducible factor 1 (HIF-1) target gene involved in the regulation of cell survival [33,34]. In the presence of mutations such as LKB1/AMPK deficiency, p53 mutation, organic cation transporter-1 (OCT1) expression or low glucose condition, metformin treatment showed cytotoxicity in cancers [35].

Studies have reported the efficacy of metformin in the treatment of TNBCs [8], however, reports suggest that higher glucose concentrations may negatively impact the anti-cancer efficacy of metformin. Increased glucose concentrations promoted breast cancer proliferation and reduced the efficiency of metformin [36]. In the current study, we hypothesized that the anti-cancer effect of metformin may be compromised in a diabetic individual who develops a triple negative form of breast cancer due to the high glucose concentrations in circulation. Additionally, reports reveal a better chemotherapeutic response with metformin when reducing the glucose available to breast cancer cells [8,36]. Metformin was found to be lethal to breast cancer cells and cancer stem cells under glucose-starved conditions [37]. Initially, we studied the effect of varying concentrations of metformin (25–10 mM) on cell proliferation in 25 mM glucose exposed TNBCs (mimics diabetes/hyperglycemia), in 5.5 mM glucose exposed TNBCs (mimics normoglycemia). We then compared the effect of 50 μM metformin (normal concentration of metformin achieved in circulation when used as an anti-diabetic drug) and a higher concentration of 2 mM metformin for its effect on apoptosis, cell-cycle, and components of the mTOR pathway under the different concentrations of glucose (25 mM and 5.5 mM) and glucose-starved conditions.

## 2. Materials and Methods

### 2.1. Chemicals, Biochemicals, Reagents, and Antibodies

Analytical grade biochemicals, reagents, and chemicals were used unless otherwise stated, including metformin (Cat # D150959) and were purchased from Sigma-Aldrich, Inc. (St. Louis, MO, USA), unless otherwise stated. Primary antibodies against pmTOR (S2448; Cat # 5536), mTOR (Cat # 2983), p4E-BP1 (T37/46; Cat # 2855), 4E-BP1 (Cat # 9644), pS6 (S235/236; Cat # 4858), pS6 (S240/244; Cat # 5364), S6 ribosomal protein (Cat # 2317) and β-actin (Cat # 3700) were purchased from Cell Signaling Technology, Inc. (Danvers, MA, USA). For western blotting, HRP linked secondary antibodies (anti-rabbit IgG, Cat # 7074 and anti-mouse IgG, Cat # 7076) were purchased from Cell Signaling Technology, Inc. (Danvers, MA, USA).

### 2.2. Cell Culture

M. D. Anderson Metastatic Breast adenocarcinoma-468 (MDA-MB-468) cells (Cat # HTB-132) and M. D. Anderson Metastatic Breast adenocarcinoma-231 (MDA-MB-231) cells (Cat # HTB-26) were purchased from American Type Culture Collection (ATCC, Manassas, VA, USA) and cultured in high glucose condition until 75% confluent for the study as previously described [33]. Passages p5–p15 were used for the study. The cells were grown in Dulbecco’s modified Eagle’s medium (Cat # 11995; Dulbecco’s Modified Eagle Media (DMEM); Invitrogen, Carlsbad, CA, USA), at 25 mM glucose concentration, supplemented with 10% FBS (Sigma-Aldrich, St. Louis, MO, USA), in a humidified atmosphere with 5% CO_2_ at 37 °C [33,38].

### 2.3. Cell Treatments

Dulbecco’s Modified Eagle Media (DMEM) (containing 25 mM glucose, here on referred to as high glucose condition or 5.5 mM glucose, here on referred to as normal glucose condition or without glucose, here on referred to as no glucose condition) was prepared with varying concentrations of metformin (at 25 μM, 50 μM, 100 μM, 250 μM, 500 μM, 1 mM, 2 mM, 5 mM, and 10 mM) from a stock 100 mM metformin solution (prepared in DMEM with no glucose (Cat # 11966). Cells exposed to media without glucose or with 5.5 mM or 25 mM glucose, in the absence of metformin were used as suitable controls. This was followed by cell proliferation/3-(4,5-dimethylthiazol-2-yl)-5-(3-carboxymethoxyphenyl)-2-(4-sulfophenyl)-2*H*-tetrazolium) (MTS) (dose response) assay. Based on the data obtained from the MTS assay, cells treated in the absence or presence of 50 μM or 2 mM metformin under the different glucose concentrations (25 mM glucose or 5.5 mM glucose or without glucose) were used for further experiments such as cell cycle analysis, apoptosis assays and western blotting.

50 μM is the maximum expected physiological concentration of metformin as a therapeutic for type 2 diabetes [33,39]. Previous research has shown that metformin is effective at 2 mM for in vitro experiments [33]. Therefore, metformin at 50 μM and 2 mM were used for experiments that followed.

### 2.4. Cell Proliferation Assay

CellTiter 96^®^ AQ_ueous_ One Solution Cell Proliferation Assay kit (Cat # G3582, Promega Corporation, Madison, WI, USA) was used to analyze cell proliferation rate as per protocol recommended in the kit. 96 well plates were used and 50,000 cells were plated in each well. The cells were at 37 °C in a CO_2_ incubator and on the third day, various dilutions of the metformin in different glucose conditions were added. The cells were treated with the drug for 72 h and following which the cells were incubated with 20 μL/well MTS reagent for 1 h. The absorbance was then read at 490 nm using a multiplate reader, CLARIOstar^®^ (BMG-LABTECH, Cary, NC, USA). Wells containing only media and MTS reagent (without cells) was used as blank for background subtraction. The quantity of formazan product as measured by absorbance at 490 nm is directly proportional to the number of living cells in culture. The cell viability was calculated as percentage of viable cells compared to untreated control (100% viable) using the equation: % Viable = Absorbance_test_/Absorbance_control_ × 100.

### 2.5. Apoptosis by Flow Cytometry

Cells were treated with different glucose conditions and metformin for 72 h and harvested by trypsinization. The cells were incubated with Annexin V fluorescein isothiocyanate (FITC) and propidium iodide (PI) at RT-25 °C for 15 min. After this incubation, the cells were washed in PBS and re-suspended in 400 μL Annexin V binding buffer (1×) supplied by the manufacturers (10×). The fluorescence was acquired using BD LSR Fortessa (BD Biosciences, San Jose, CA, USA) with 488 nM laser for excitation and emission with two filters 585/15 and 610/20 for Annexin V FITC and PI respectively. Data was obtained using FACSDiva v6.3 (BD Biosciences, San Jose, CA, USA). PMT voltages were defined on untreated unstained cells. Single stained samples were used for compensation and for gating. 10,000 events were recorded for each sample. Annexin V conjugated with FITC binds to apoptotic cells. PI is a DNA binding dye that stains dead and damaged cells. Thus, four distinct cell populations are observed and quantified (indicated by four quadrants (Q) in the graph) which are (1) early apoptotic (FITC Annexin V positive and PI negative); (2) late apoptotic (FITC Annexin V and PI positive); (3) live (FITC Annexin V and PI negative); and (4) necrotic (FITC Annexin V and PI positive) [38]. Positive control for Annexin V FITC staining was 72 h staurosporine treatment. Cells were heated at 56 °C for 3 min as positive control for PI. After the completion of the treatment protocols cells were harvested and stained with 5 μL each of PI and Annexin V FITC for the treated cells. (Cat# 556547, BD Pharmingen™)

### 2.6. Cell Cycle Analysis Using Propidium Iodide Staining by Flow Cytometry

After the completion of the treatment protocols cell culture media was collected and centrifuged at 3000 rpm for 5 min to retrieve all the dead cells while the adherent cells were trypsinized and centrifuged at 3000 rpm for 5 min. Cells from the dead and adherent groups were pooled and suspended in 1 mL DPBS. This 1 mL of cell suspension was added dropwise to 2.5 mL of absolute alcohol (final concentration 70%) and stored overnight at −20 °C. The following day cells were treated with 500 μL of PI solution (containing 50 μg/mL of PI, 0.1 mg/mL of RNase and 0.1% Triton × 100 in PBS) for 45 min at 37 °C. After washing with DPBS at 3000 rpm for 5 min, they were analyzed on BD LSR Fortessa (BD Biosciences, San Jose, CA, USA). The results were analyzed, and gating was performed on BD FACSDiva v6.3 (BD Biosciences, San Jose, CA, USA) software.

### 2.7. Protein Isolation and Total Protein Estimation

Following treatment, the protein was harvested, as previously described [33,39], using a lysis buffer of composition 1× RIPA buffer (Cat # R0278 Sigma-Aldrich, St. Louis, MO, USA), containing 10 μL/mL of Halt protease and phosphatase inhibitor (Cat # 78440, Thermo-Fisher Scientific, Waltham, MA, USA) and 10 μL/mL of 0.5 M EDTA pH = 8.0 (Cat # 15575020, Thermo-Fisher Scientific, Waltham, MA, USA). The cell extracts were lysed 8–10 times through a 23 g needle on a 3 mL syringe maintained on ice for 30–45 min with intermittent vortexing and was then centrifuged at 13,000 rpm for 10 min at 4 °C using the Eppendorf 5417R refrigerated centrifuge (Eppendorf, Hauppauge, NY, USA). The supernatant (protein lysate) was used for immunoblotting. The total protein concentration of each of the samples was estimated as per kit protocol using the Bio-Rad DC protein assay kit (Cat # 5000112, Bio-Rad, Hercules, CA, USA). The absorbance was then read at 750 nm using a 96-well ‘EnVision 2104 multilabel’ plate reader (PerkinElmer, Inc., Waltham, MA, USA).

### 2.8. Immunoblotting

SDS-Polyacrylamide Gel Electrophoresis (SDS-PAGE) followed by immuno-blotting was used to detect the levels of pmTOR (S2448), mTOR, p4E-BP1 (T37/46;), 4E-BP1, pS6 (S235/236), pS6 (S240/244), S6 ribosomal protein and β-actin as previously described [33]. The protein bands after western blotting were detected using enhanced chemiluminescence reagent (Sigma-Aldrich, Inc., St. Louis, MO, USA) and imaged on a Geliance P600 gel documentation system (PerkinElmer, Inc., Waltham, MA, USA). The blot used for the total protein was stripped to detect and probe the respective phospho-protein form. Loading control used was β-actin.

### 2.9. Statistical Analysis

All data were analyzed with the statistical software GraphPad Prism 7.0 (GraphPad Software, Inc., San Diego, CA, USA). Data are presented as mean ± SEM. Statistical analysis was performed using Student’s *t*-test or one-way analysis of variance (ANOVA). Post-hoc comparisons between groups after ANOVA were performed by Tukey’s multiple comparison tests. ‘*p*’ values less than 0.05 were considered to be statistically significant [33].

## 3. Results

### 3.1. Effect of Metformin on Cell Proliferation in MDA-MB-231 and MDA-MB-468 Cells Exposed to 25 mM Glucose and 5.5 mM Glucose Conditions

The effect of different concentrations of metformin on TNBC cell proliferation varies depending on the glucose concentration in the microenvironment and cell type.

**Figure 1:**
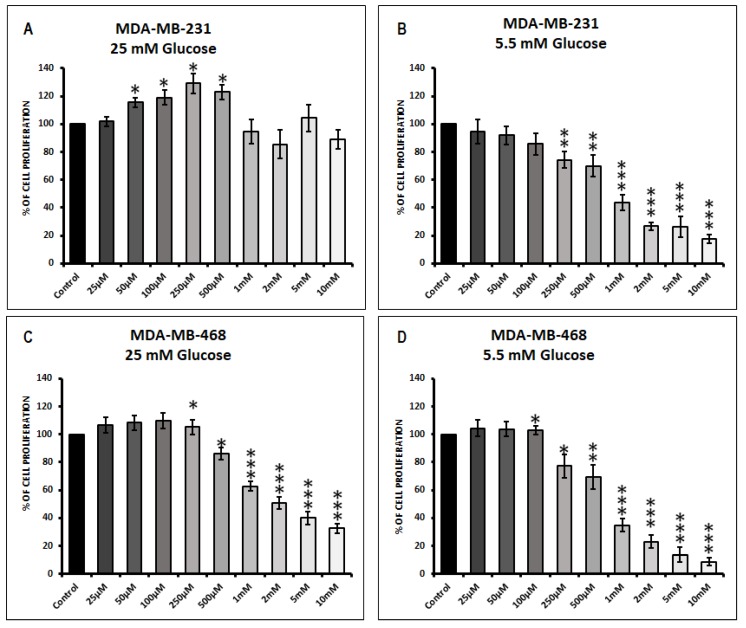
Response of MDA-MB-231 and MDA-MB-468 to 72 h treatment of metformin cultured in varying glucose conditions. Cell proliferation upon metformin treatment was measured using 3-(4,5-dimethylthiazol-2-yl)-5-(3-carboxymethoxyphenyl)-2-(4-sulfophenyl)-2*H*-tetrazolium) (MTS) assay in (**A**) MDA-MB-231 cells at 25 mM glucose condition, (**B**) MDA-MB-231 cells at 5.5 mM glucose condition, (**C**) MDA-MB-468 cells at 25 mM glucose condition and (**D**) MDA-MB-468 cells at 5.5 mM glucose condition. Data represent the mean ± standard error of mean of 5–7 independent experiments. * *p*-value < 0.05, ** *p*-value < 0.01 and *** *p*-value < 0.001 was considered to be significant with respect to untreated control.

MDA-MB-231 cells in 25 mM glucose condition when treated with lower concentrations of metformin (50, 100, 250, and 500 μM) significantly increased cell proliferation when compared to its untreated control (* *p*-values < 0.05). Higher concentrations of metformin (1, 2, 5, and 10 mM) showed a decreasing trend in cell proliferation was not significant compared to untreated control in 25 mM glucose exposed MDA-MB-231 cells (Figure 1A). However, in 5.5 mM glucose exposed MDA-MB-231 cells metformin concentrations (250 μM, 500 μM, 1 mM, 2 mM, 5 mM, and 10 mM) showed a significant decrease in MDA-MB-231 cell proliferation compared to its untreated control (Figure 1B; ** *p*-value <0.01, *** *p*-value < 0.001).

In 25 mM glucose exposed MDA-MB-468 cells treatment with higher concentrations of metformin (500 μM, 1 mM, 2 mM, 5 mM, and 10 mM) significantly decreased cell proliferation when compared to untreated control (Figure 1C; *** *p*-value < 0.001). Lower concentrations of metformin (50, 100, 250, and 500 μM) increased cell proliferation in 25 mM glucose exposed MDA-MB-468 cells but were not statistically significant. Under normoglycemic (5.5 mM glucose) concentrations (250 μM, 500 μM, 1 mM, 2 mM, 5 mM, and 10 mM) showed a significant decrease in MDA-MB-468 cell proliferation compared to its untreated control (Figure 1D; ** *p*-value < 0.01, *** *p*-value < 0.001).

### 3.2. Effect of Metformin on Apoptotic Cell Death in MDA-MB-231 and MDA-MB-468 Cells Exposed to Different Glucose (25 mM, 5.5 mM, and Zero Glucose/Glucose-Starved) Conditions

The effect of different concentrations of metformin on TNBC cell apoptosis varies depending on the glucose concentration in the microenvironment and cell type.

**Figure 2:**
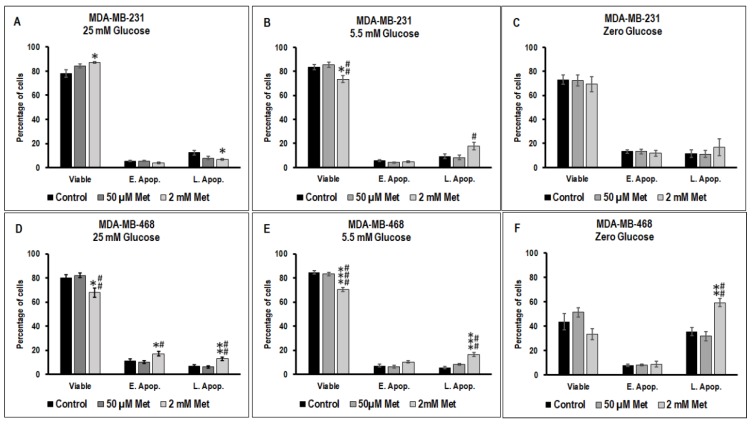
The effect of metformin (50 μM and 2 mM) on apoptosis in 25 mM, 5.5 mM, and zero glucose/glucose-starved conditions in MDA-MB-231 and MDA-MB-468 cells was measured using flow cytometry with fluorescein isothiocyanate (FITC) tagged Annexin V and propidium iodide (PI) in (**A**) MDA-MB-231 cells at 25 mM glucose condition, (**B**) MDA-MB-231 cells at 5.5 mM glucose condition, (**C**) MDA-MB-231 cells at zero glucose/glucose-starved condition, (**D**) MDA-MB-468 cells at 25 mM glucose condition, (**E**) MDA-MB-468 cells at 5.5 mM glucose condition, and (**F**) MDA-MB-468 cells at zero glucose/glucose-starved condition. ‘E. Apop.’ indicates early apoptotic events and ‘L. Apop.’ indicates late apoptotic events. Data represent the mean ± standard error of mean of 5–7 independent experiments. *p*-value < 0.05, ** *p*-value < 0.01, and *** *p*-value < 0.001 was considered to be significant with respect to untreated control and # *p*-value < 0.05, ## *p*-value < 0.01, and ### *p*-value < 0.001 was considered to be significant with respect to 50μM metformin treated cells.

In 25 mM glucose exposed MDA-MB-231 cells, treatment with 2 mM metformin significantly increased viable cell population (* *p*-value < 0.05) and decreased the late apoptotic populations (* *p*-value < 0.05) (Figure 2A). Whereas in 5.5 mM glucose exposed MDA-MB-231 cells, treatment with 2 mM metformin significantly reduced viable MDA-MB-231 population when compared to untreated control (* *p*-value < 0.05) and 50 μM metformin treated cells (## *p*-value < 0.01) and significantly increased late apoptotic population when compared to 50 μM metformin treated MDA-MB-231 cells (# *p*-value < 0.05) (Figure 2B). There was a marked increase (not significant) in apoptosis in 2 mM metformin treated glucose-starved MDA-MB-231 cells when compared to untreated and 50 μM metformin treated glucose-starved cells (Figure 2C).

In 25 mM glucose exposed MDA-MB-468 cells, treatment with 2 mM metformin significantly decreased the viable population when compared to untreated control (* *p*-value < 0.05) and 50 μM metformin treated cells (## *p*-value < 0.01) (Figure 2D). A significant increase in early apoptotic cell population (* *p*-value < 0.05; when compared to untreated control, # *p*-value < 0.05; when compared 50 μM metformin treated cells) was observed in 2 mM metformin treated MDA-MB-468 cells maintained in 25 mM glucose condition (Figure 2D). Additionally, a significant increase in late apoptotic cell population (** *p*-value < 0.01; when compared to untreated control, ## *p*-value < 0.01; when compared 50 μM metformin treated cells) was observed in 2 mM metformin treated MDA-MB-468 cells maintained in 25 mM glucose condition (Figure 2D).

Similarly, in 5.5 mM glucose condition exposed MDA-MB-468 cells treatment with 2 mM metformin significantly decreased the viable population when compared to untreated control (*** *p*-value < 0.001) and 50 μM metformin treated cells (### *p*-value< 0.001) (Figure 2E). A significant increase in late apoptotic cell population (*** *p*-value < 0.001; when compared to untreated control, ## *p*-value < 0.01; when compared 50 μM metformin treated cells) was observed in 2 mM metformin treated MDA-MB-468 cells maintained in 5.5 mM glucose condition (Figure 2E).

Zero glucose/glucose-starved condition decreased the overall viability of MDA-MB-468 cells when compared to 25 mM glucose exposed and 5.5 mM glucose exposed MDA-MB-468 cells (Figure 2F). Treatment with 2 mM metformin in glucose-starved MDA-MB-468 cells significantly increased the late apoptotic cell population when compared to untreated control (** *p*-value < 0.01) and 50 μM metformin treated cells (## *p*-value < 0.01) (Figure 2F).

### 3.3. Effect of Metformin on Cell Cycle in MDA-MB-231 and MDA-MB-468 Cells Exposed to Different Glucose (25 mM, 5.5 mM, and Zero Glucose/Glucose-Starved) Conditions

**Figure 3:**
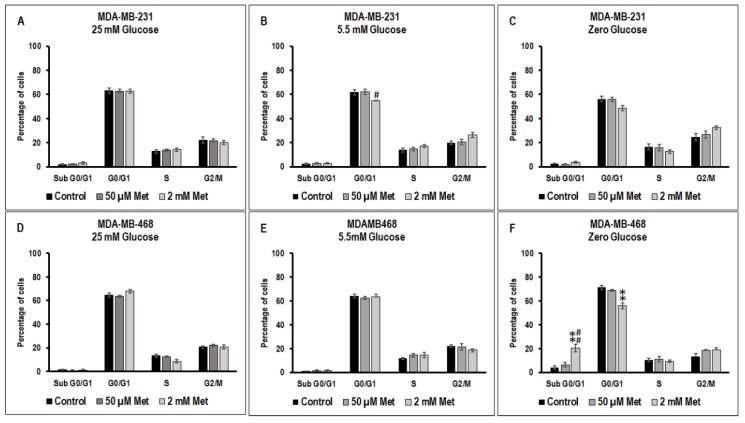
The effect of metformin (50 μM and 2 mM) on the cell cycle in 25 mM, 5.5 mM, and zero glucose/glucose-starved conditions in MDA-MB-231 and MDA-MB-468 cells was measured using flow cytometry with fluorescein isothiocyanate (FITC) tagged Annexin V and propidium iodide (PI) in (**A**) MDA-MB-231 cells at 25 mM glucose condition, (**B**) MDA-MB-231 cells at 5.5 mM glucose condition, (**C**) MDA-MB-231 cells at zero glucose/glucose-starved condition, (**D**) MDA-MB-468 cells at 25 mM glucose condition, (**E**) MDA-MB-468 cells at 5.5 mM glucose condition, and (**F**) MDA-MB-468 cells at zero glucose/glucose-starved condition. Data represent the mean ± standard error of mean of 5–7 independent experiments. ** *p*-value < 0.01 was considered to be significant with respect to untreated control and # *p*-value < 0.05 and ## *p*-value < 0.01 was considered to be significant with respect to 50 μM metformin treated cells.

There were no significant changes in the different phases of the cell cycle between untreated, 50 μM metformin treated and 2 mM metformin treated MDA-MB-231 cells exposed to 25 mM glucose. In 5.5 mM glucose condition, treatment with 2 mM metformin significantly decreased the MDA-MB-231 cell population at G_0_/G_1_ phase with respect to 50 μM metformin treated cells (# *p*-value < 0.05) (Figure 3B). There was a slight increase (not significant) in cells in the subG_0_/G_1_ in 2 mM metformin treated glucose-starved MDA-MB-231 cells when compared to non-treated and 50 μM metformin treated cells in zero glucose/glucose-starved condition (Figure 3C).

In both 25 mM and 5.5 mM glucose exposed MDA-MB-468 cells, there were no significant changes with metformin treatment compared to untreated control (Figure 3D). However, in the zero glucose/glucose-starved condition, treatment with 2 mM metformin significantly increased the MDA-MB-468 cell population at subG_0_/G_1_ phase with respect to glucose-starved untreated controls (** *p*-value < 0.01) and glucose-starved 50μM metformin treated cells (## *p*-value < 0.01) and reduced the cell population at G_0_/G_1_ phase with respect to glucose-starved untreated controls (** *p* values < 0.01).

### 3.4. Effect of Metformin on the mTOR Pathway in MDA-MB-231 and MDA-MB-468 Cells Exposed to Different Glucose (25 mM, 5.5 mM, and Zero Glucose/Glucose-Starved) Conditions

The mTOR pathway is known to play a key role supporting the rapid proliferation of breast cancer cells and therefore we studied the effects of treatment with metformin on the modulation of mTOR and its downstream targets.

**Figure 4:**
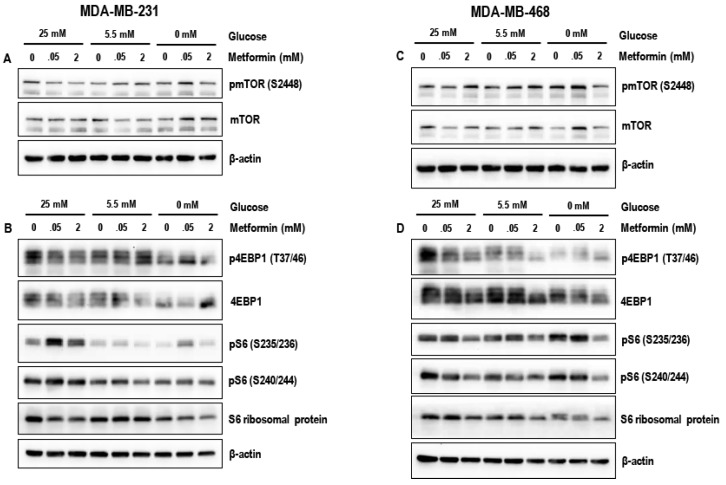
Representative western blots (**A**) and (**B**) show the effect of metformin (50 μM and 2 mM) in 25 mM glucose, 5.5 mM glucose, and zero glucose/glucose-starved conditions on the levels of pmTOR (S2448), mTOR, p4EBP1 (T37/46), 4EBP1, pS6 (S235/236), pS6 (S240/244), and S6 ribosomal protein in MDA-MB-231 cells. Representative western blots (**C**) and (**D**) show the effect of metformin (50 μM and 2 mM) in 25 mM glucose, 5.5 mM glucose, and zero glucose/glucose-starved conditions on the levels of pmTOR (S2448) and mTOR, p4EBP1 (T37/46), 4EBP1, pS6 (S235/236), pS6 (S240/244), and S6 ribosomal protein in MDA-MB-468 cells. β-actin was used as the loading control. Data represented is from 3–4 independent experiments.

Treatment with 50 μM metformin for 72 h did not markedly alter the levels of key proteins of the mTOR pathway pmTOR (S2448), p4EBP1 (T37/46), pS6 (S235/236), and pS6 (S240/244) under the different glucose conditions (25 mM, 5.5 mM and glucose-starved) in both MDA-MB-231 and MDA-MB-468 cells (Figure 4A–D) when compared to the untreated controls. In contrast, treatment with 2 mM metformin for 72 h markedly reduced the levels of pmTOR (S2448), p4EBP1 (T37/46), pS6 (S235/236), and pS6 (S240/244) in glucose-starved MDA-MB-231 and MDA-MB-468 cells when compared to metformin (2 mM treated) 25 mM glucose and 5.5 mM glucose exposed cells (Figure 4A–D). The data suggests that treatment with metformin (2 mM) is most effective in inhibition of the mTOR pathway under glucose-starved conditions. Alternatively, since glucose-starvation cannot be clinically attained, studies using metformin in combination with glycolytic inhibitors such as 2-deoxyglucose may be necessary to test whether similar results can be obtained.

**Figure 5:**
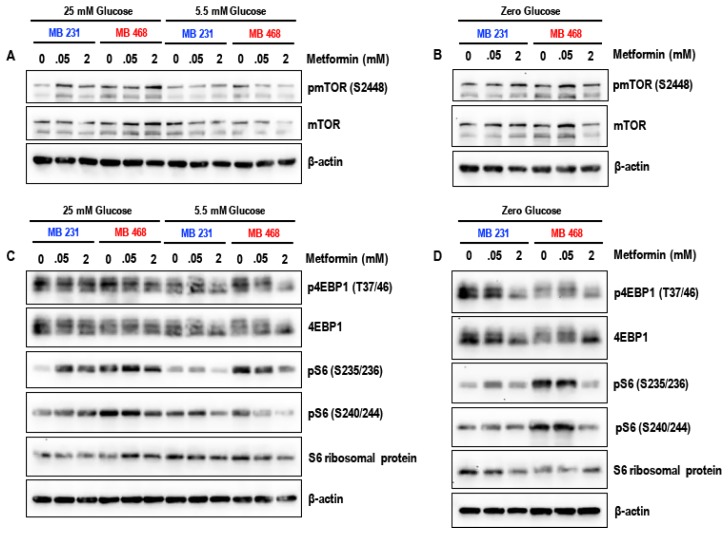
Side-by-side comparison between MDA-MB-231 and MDA-MB-468 cells. Representative western blots (**A**) show the effect of metformin (50 μM and 2 mM) in 25 mM glucose and 5.5 mM glucose on the levels of pmTOR (S2448) and mTOR in MDA-MB-231 and MDA-MB-468 cells, (**B**) show the effect of metformin (50 μM and 2 mM) in zero glucose/glucose-starved condition on the levels of pmTOR (S2448) and mTOR in MDA-MB-231 and MDA-MB-468 cells, (**C**) show the effect of metformin (50 μM and 2 mM) in 25 mM glucose and 5.5 mM glucose on the levels of p4EBP1 (T37/46), 4EBP1, pS6 (S235/236), pS6 (S240/244), and S6 ribosomal protein in MDA-MB-231 and MDA-MB-468 cells, and (**D**) show the effect of metformin (50 μM and 2 mM) in zero glucose/glucose-starved condition on the levels of p4EBP1 (T37/46), 4EBP1, pS6 (S235/236), pS6 (S240/244), and S6 ribosomal protein in MDA-MB-231 and MDA-MB-468 cells. β-actin was used as the loading control.

The comparison in the levels of pmTOR (S2448), p4EBP1 (T37/46), pS6 (S235/236), and pS6 (S240/244) between the MDA-MB-231 and MDA-MB-468 cells (Figure 5A–D) indicate that the MDA-MB-231 cells are resistant to the effects of the glucose starvation and may be able to maintain mTOR pathway mediated protein synthesis under glucose-starved conditions when compared to MDA-MB-468 cells.

## 4. Discussion

The present study demonstrates that an increase in glucose concentration reduces the efficacy of metformin thereby requiring metformin at higher concentrations to exhibit its anti-cancer effects. Physiological metformin concentrations (50–100 μM) in the presence of higher glucose concentrations also seemed to promote cancer cell proliferation in MDA-MB-231 cells. Apoptotic effects of metformin observed in lower concentrations of glucose are diminished when glucose concentration is increased. The apoptotic effects in glucose-starved conditions can be corroborated with our data from cell cycle analysis showing an increase in the number cells at sub-G_0_/G_1_ phase with metformin (2 mM) treatment. Our results indicate that the anti-cancer effects of metformin treatment in glucose-starved conditions were more pronounced in MDA-MB-468 cell type than in MDA-MB-231 cell type.

Cancer cells proliferate at a higher rate in the abundance of glucose (diabetes) through aerobic glycolysis (Warburg effect) in comparison to normal cells [3]. While glucose restriction/starvation should reduce cancer proliferation [1], in several cases these cancer cells tend to derive the energy for necessary cell processes and proliferation through alternative pathways [40]. Multiple reports have supported the connection between metformin, diabetes, and breast cancer [21,41,42]. Treatment with metformin has been linked (1) to a lower incidence of breast cancer in diabetic patients, (2) to improved response to chemotherapy and radio-therapy and better prognosis, and (3) to improved overall survival in diabetic cancer patients [4,43,44]. Reports suggest that incidence of invasive breast cancer was lower in diabetic patients taking metformin when compared to those who took other anti-diabetic medications, like sulfonylureas (e.g., Glipizide, Glibenclamide) [45]. In another report, diabetic women who were taking metformin had a better response to chemotherapy and lower incidence of certain types of tumors [46]. A “complete response” (no remaining cancer in the affected breast or lymph node(s) was more likely in diabetic breast cancer patients that received chemotherapy and metformin when compared to those who received chemotherapy alone [47].

In a diabetic patient on a metformin treatment regimen, the therapeutically achievable level of metformin in the plasma is around 50–100 μM [39,48,49]. However, a vast majority of in vitro studies show the efficacy of metformin as an anti-cancer agent at very high concentrations (>5 mM). This may be due to the high glucose concentrations used in the culture most cancer cell lines. The presence of glucose at high concentrations reduced the anti-neoplastic efficacy of metformin and therefore a much higher concentration of metformin (>5 mM) was required to achieve cytotoxic/anti-cancer effect in the presence of high glucose concentrations [33,37,50].

Given the aggressiveness, invasiveness, metastatic potential, poor prognosis, and lack of therapeutic targets for TNBCs it is possible that using metformin in combination with existing chemotherapeutic agents will prove efficient in the treatment of such cancers. A synergistic increase in cancer cell apoptosis was observed when metformin was used in combination with chemotherapeutic drugs. Metformin treatment also re-sensitized cells and made them susceptible to classical anti-cancer drugs thus improving the efficacy of cancer treatment by reprogramming the altered cancer cells’ metabolism and thus can help overcome chemotherapeutic and multidrug resistance in breast cancer cells [4,51,52]. However, in a diabetic condition, management of diabetes and maintaining glucose homeostasis may be crucial in order to achieve the potential benefit of metformin when used as an anti-cancer agent.

Our data from proliferation assays show a biphasic effect of metformin that varies not only with concentration but also varies with different cell types when using the same concentration. Biphasic effect or ‘Hormesis’, is defined as a dose-response relationship phenomenon characterized by low-dose stimulation and high-dose inhibition independent of the chemical/physical agent or any biological model [53]. This effect emphasizes the impact of different dosages/concentrations in the assessment and selection of compounds as chemotherapeutic agents. [23,53]. The biphasic effect is prominent in MDA-MB-231 cells when low concentration of metformin treatment increased cell viability/proliferation at high glucose conditions (25 mM) while similar metformin concentrations reduced cell viability in normal glucose conditions, indicating a protective effect of metformin at higher glucose conditions. MDA-MB-468 showed significant reduction in cell population with metformin (1 mM and above) treatment in high glucose (25 mM) conditions whereas MDA-MB-231 cells showed no response to metformin with the same levels of glucose. Low concentrations of metformin have been shown to confer protection to endothelial cells exposed high-glucose conditions from apoptosis and senescence through the activation of Sirtuin 1 and depletion of micro-RNA 34a (miRNA-34a) [39,54]. Low doses of metformin also inhibit the generation of reactive oxygen species and negates oxidative stress [39,54]. Studies are warranted to address and explain the molecular mechanisms of the protective or pro-proliferative effect of low doses of metformin under high glucose concentrations. Such studies are critical since prescribing metformin to diabetic breast cancer patient with an MDA-MB-231 type of cancer may support the growth and progression of cancer in a scenario where hyperglycemia is not properly managed.

Metformin induces apoptosis in MDA-MB-468 cells [55] in a concentration dependent manner and through a mitochondrial pathway in MDA-MB-231 cells [56]. Treatment with metformin and without glucose has shown reduced viability of MDA-MB-231 cells [57]. According to our data, at 72 h post-treatment, the apoptotic effect of metformin is more significant in MDA-MB-468 cells at lower glucose concentrations than MDA-MB-231 cells.

For both MDA-MB-231 and MDA-MB-468 cell lines, our results from the apoptosis assay suggest that higher concentrations metformin reduces viability at lower glucose conditions. The same biphasic effect can be inferred thorough the apoptosis assay where we observed a higher viable population in MDA-MB-231 cells with 25 mM glucose conditions. Thus, apoptotic effects of metformin are more significant at 5.5 mM glucose than 25 mM glucose.

Cell cycle arrests at G_0_/G_1_ with very high concentrations of metformin were reported in various cancers such as melanoma [58,59], glioblastoma and prostate cancer. Our cell cycle analysis revealed a G_2_/M phase arrest with 2 mM metformin only at lower glucose conditions.

The studies were performed in two different types of TNBCs, MDA-MB-231 cells (and MDA-MB-468 cells). The MDA-MB-231 (mesenchymal stem cell-like; MSL) cells and MDA-MB-468 cells (basal like-1; BL-1) differ greatly in morphology and at the molecular level having differential gene expression and distinct gene ontologies [60,61]. Interestingly, lower concentrations of metformin (50, 100, 250, and 500 μM) significantly increased cell proliferation in 25 mM glucose exposed MDA-MB-231 cells, an effect which was not observed in MDA-MB-468 cells, indicating that the effective concentration of metformin when used as anti-cancer drug in TNBCs may have to be determined based on cell type and blood glucose concentration. These variations between the TNBC subtypes could be a possible explanation for the difference in response to identical glucose and metformin treatment.

We have also noted that the efficacy of metformin, with respect to decreasing TNBC cell proliferation, increasing apoptosis and inhibiting the mTOR pathway (pro-survival and protein synthesis pathway activated in cancer cells), significantly increased under zero glucose/glucose-starved conditions. This is in accordance with similar reports that suggest metformin becomes lethal to cancer cells under conditions of glucose-starvation/withdrawal [37]. Glucose starvation, while feasible in an in vitro setup, is practically impossible in a clinical scenario. Therefore, in order to mimic this condition of glucose-starvation and metformin treatment, it is possible that using glycolytic/metabolic inhibitors in combination with metformin should cause a similar anti-cancer effect. Several studies have reported that using a combination of the glycolytic inhibitor, 2-deoxyglucose and metformin was found to be efficient and have shown synergistic effects in the treatment of cancers when compared to using 2-deoxyglucose or metformin alone [33,62,63].

## 5. Conclusions

The present study demonstrates that MDA-MB-231 and MDA-MB-468 have a difference in the response to metformin treatment, which may depend on the amount of glucose available to the cells. Although MDA-MB-231 cells show apoptosis with 2 mM metformin, the effect was not significant at higher concentration of glucose, whereas MDA-MB-468 cells respond to metformin treatment even in the presence of high (25 mM) glucose concentrations.

We have thus shown that efficiency of metformin anti-cancer drug may be inversely related to the amount of glucose available in the medium. The data also show that it is critical to manage hyperglycemia and diabetes before metformin treatment especially depending on the subtype of TNBC to benefit from the anti-cancer effect of metformin. In case of TNBCs, the chemotherapeutic effect of metformin could either be via the regulating glucose homeostasis or by directly targeting pro-survival pathways in cancer cells [34,57]. Moreover combining metformin with an established regimen of cancer therapeutic agents could possibly have synergistic anti-cancer effects while lowering the dosage required to achieve these effects with maximum efficacy [64].
